# Supplementary material for: Splice-Junction-Based Mapping of Alternative Isoforms in the Human Proteome
Source: Cell Rep. Author manuscript; Available in PMC 2020 Jan 15. (PMC6961840; doi:10.1016/j.celrep.2019.11.026)

A

Predicted sequence disorder and sequence features of P46976

Peptide: DTCSYVNVEDVSGAISHLSLGEIPAMAQPFVSSEERK Junction: sp|P46976|GLYG_HUMAN|ENSG00000163754|SE2|17403|chr3|149024272|149026502|+1|r340|T1 TrNovel: FALSE

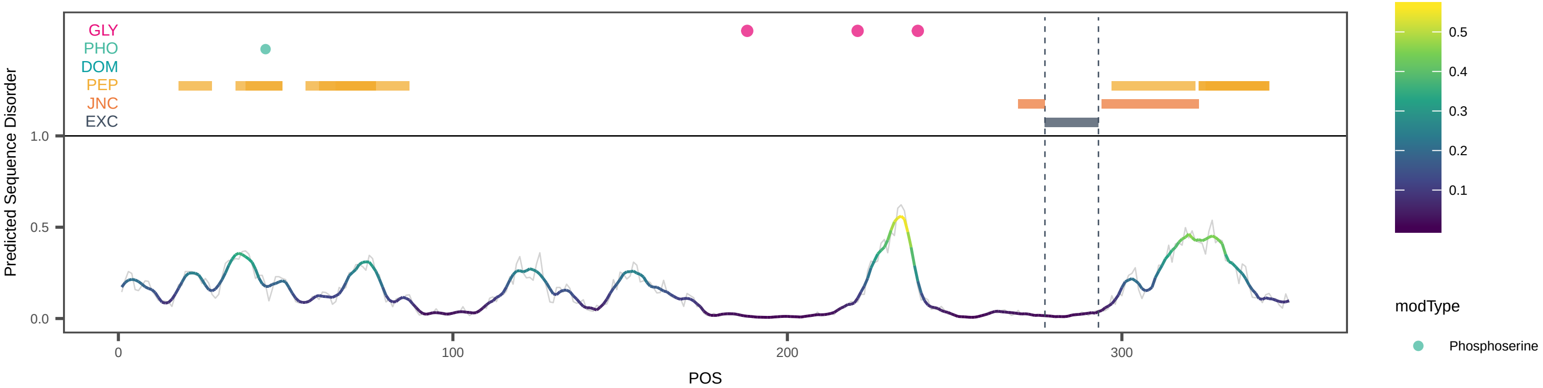

B

Distribution of sequence disorder in excised vs. mapped and non−excised regions of protein

M−W P−value vs. mapped: 4.85e−11 vs. non−excised: 1.28e−07

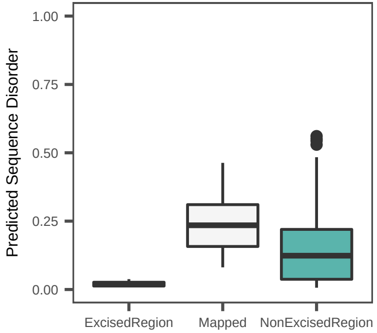

C

Enrichment of phosphosites in skipped exons spanned by identified splice junction

Fisher's exact test P: 1

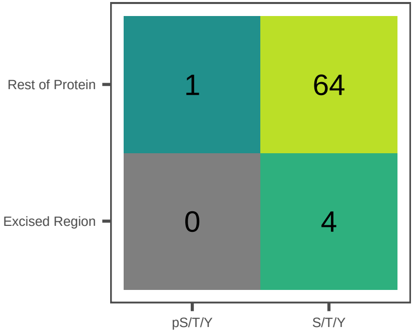

Supplement: 3 [file NIHMS1546469-supplement-3.zip › DF2/PXD0010154/Esophagus-135-P46976-DTCSYVNVEDVSGAISHLSLGEIPAMAQPFVSSEERK.pdf]
